# Supplementary material for: Dek504 Encodes a Mitochondrion-Targeted E+-Type Pentatricopeptide Repeat Protein Essential for RNA Editing and Seed Development in Maize
Source: Int J Mol Sci. 2022 Feb 24;23(5):2513. doi: 10.3390/ijms23052513 (PMC8910059; doi:10.3390/ijms23052513)
Supplement: Supplementary file 1 [file ijms-23-02513-s001.zip › Supplemental Figure.pdf]

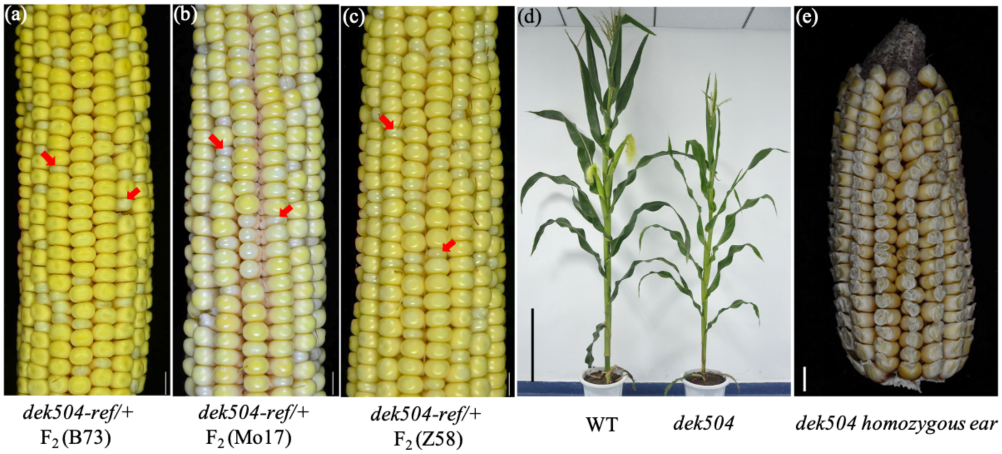


**Figure S1.** The phenotype of *dek504* in different hybridization backgrounds and phenotypes of *dek504* alleles. (**a**–**c**) A selfed ear segregates 3:1 for the wild-type (WT) and *dek504* mutant kernels (arrows). (**a**–**c**) belong to B73, Mo17 and Z58 backgrounds respectively. (**d**) The phenotype in the reproductive stages of *dek504* and WT ('B73') plants in the field respectively. (**e**) A homozygous ear of *dek504*. (**a**–**e**) Scale bar = 1 cm. (**d**) Scale bar = 0.5 m.

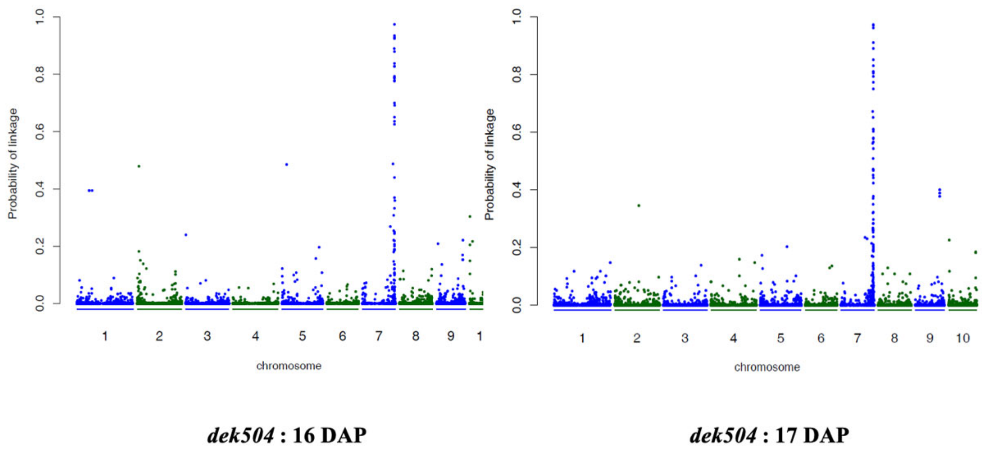


**Figure S2.** BSR-seq mapping of *dek504*. Mapping of *dek504* gene was performed using RNA sequencing of plant pools of wild type (WT) and *dek504-ref* kernels (pericycle removed) collected from the same ear. The probability of linkage of each SNP (Single Nucleotide Polymorphism) with the causal gene was determined by a Bayesian approach and plotted versus genomic coordinate of the SNP.

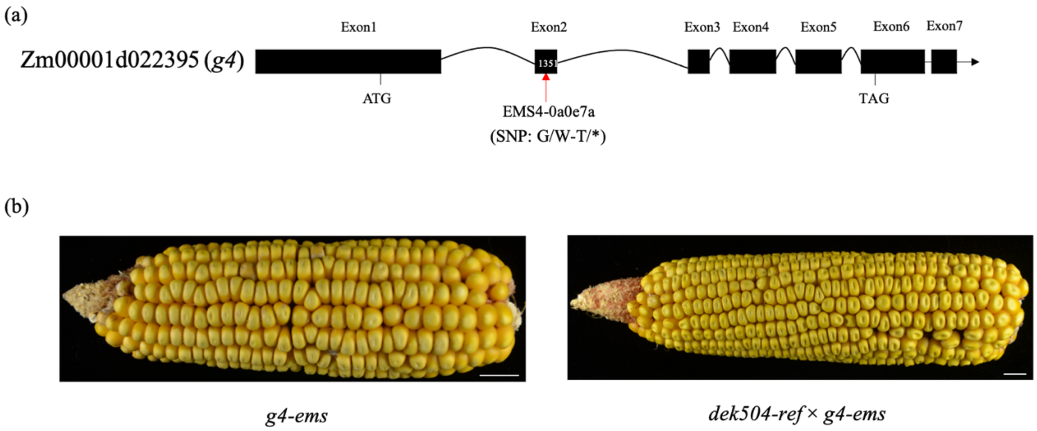


**Figure S3.** Allelic tests between *dek504-ref* heterozygotes and *g4*-ems homozygous mutants.

(**a**) Structure and mutation site of the *Zm00001d022395* gene. The red arrow denotes the mutation site of *Zm0001d022395*. Lines represent introns and black boxes represent exons. (**b**) Crossed ear between *dek504-ref* heterozygotes and *g4-ems* homozygous display WT phenotype. Bar, 1cm.

WT:

ATGCATTCCGCCGCCTCCACCAATACCACCGCCTTTGCCGCCGCCGCGCTCTCTACGCT
CCTGTCGCGCTGCCGTTCCCTCGCCTCCGTCAAGCAGCTCCATGCCCACATCCTCGCAC
GTTCAAACAGCCCATTCCCCTACAATCATTTCCTCTCCAAACTCCTCTCCCTCTCCTGCT
CCGCCTCTGCCTCCGCCGCCAACTATGCCCTCCTCCTCCTCTCCTCCCACCCGGCGCCC
ACCGCCTTCACCTACAATGTCACAATCCGCTTATTCGCCTCATCGCGCCCGGACACCTC
CCTTCGCCTCTTCCTCAGCATGCTCCGCGCCGAGGTCCGCGTCCGACCCGACGCATAC
ACCCTCCCTTTCCTCCTCCTCGCCGCGGCGCGCTGCCCGGCACCGGCCCTCGCCCGGT
CCGCCCATGCTCTCCTCGAGAAGATCGGTCTCGGTGACCACGACCACACCGTCCACTC
CCTCATCACCATGTACTCCTGCCTTGATGATCACCTCGCCGCGCGCAGGGTGTTCGACG
GAATTTCCCACCGGGATGTCGTCTCTTGGAACTCGATGGTGAAGGCATACGAACGGGC
AGGGATGGTGGCCGAGGTTGAAGGGATGTTGCGGTCGATGGTCACCGAGGGCGCAGT
GGCACCCAATGGGGTGACTCTGGCGGTTGTGCTCACAGCTTGCAGGGACGCTGGCAAT
TTGGTGCTTGGGAGGTGGGTGGAGGAGTGGGTGAGGTCTGCGGGGATGGAGGTGGAC
TCGCTCATTGGGTCAGCACTTGTGGGGATGTATGAGAAGTGTGGAGAGATGGCAGAGG
CACGGCGTGTGTTTGATGGCATCAGACACAAGGATGTCGTGGCATGGAATGCCATGAT
CACCGGGTATGCGCAAAATGGCATGTCAAATGAAGCGATAGCCTTGTTTCACAGCATG
AGGGAAGCTGGGCTGTGTCCGGACAAGATAACCTTGGTCGGTGTCCTCTCATCCTGCG
CAGCAGTTGGTGCACTGAAGCTTGGAGTTGAATTGGATACGTATGCCATGCGCAGAGG
CCTCTACAACAATGTCTATGTAGGAACAGCCTTAGTGGACATGTATGCTAAGTGTGGAG
ATCTTGAGAAAGCCACGCATGTTTTTGGGAAGATGCCATTCAAAAATGAAGCCTCATG
GAATGCATTAATCTGTGGACTTGCCTTCAACGGTCGAGGCTATGATGCCATTCAGCAGT
TTGAACTAATGAGAGATGAGAAAGGACTCCAACCAGATGATATCACATTCATAGGTGT
GCTTTCGGCTTGTGTACATGCTGGGCTACTCGAGTATGGCCGCCAGTTGTTCAATTCTC
TGACACCTGTTTTTAAGATCATTCCTAGAATTGAGCACTACTCCTGCATTGTTGATTTGT
TGGCACGTGCTGGTCATTTAGAAGAAGCATGGGATTTTATTGAGGAAATGCCTGGCAA
GGTAGATGCCGTCATGTTAGGCGCTTTGCTTGCTGCTTGCCGGAAATGCAAGAACACT
GAGGTTGGTGAGAGGGTCATCAATAGGATTATGAAGCTGGAGCCATCAAACTCGTGGA
ACTACGTGGTGTCATCCAAGATTTATGCAACCTCAGATAAAATGGATGAATCTGCAAGG
ATGATAGGGCTAATGAGGGAGAGGGGTGTCAACAAGACTCCAGGGTGCAGCTGGGTT
GAGGTTAATGGAAAAGTTCTTGAATTCTATGCAAGCACTGAACCACAGCATGGTGCGG
AAGATATGTATCAACTCATGGGCGTACTGGTAAATGAGATGAGACTAAAGGGATATGTT
CCAAACCTTGATCTGGTGTAG

*dek504-ref*:

ATGCATTCCGCCGCCTCCACCAATACCACCGCCTTTGCCGCCGCCGCGCTCT
CTACGCTCCTGTCGCGCTGCCGTTCCCTCGCCTCCGTCAAGCAGCTCCATG
CCCACATCCTCGCACGTTCAAACAGCCCATTCCCCTACAATCATTTCCTCTC
CAAACTCCTCTCCCTCTCCTGCTCCGCCTCTGCCTCCGCCGCCAACTATGCC
CTCCTCCTCCTCTCCTCCCACCCGGCGCCCACCGCCTTCACCTACAATGTCA
CAATCCGCTTATTCGCCTCATCGCGCCCGGACACCTCCCTTCGCCTCTTCCT
CAGCATGCTCCGCGCCGAGGTCCGCGTCCGACCCGACGCATACACCCTCCC
TTTCCTCCTCCTCGCCGCGGCGCGCTGCCCGGCACCGGCCCTCGCCCGGTC
CGCCCATGCTCTCCTCGAGAAGATCGGTCTCGGTGACCACGACCACACCGT

[]TCGGCACCCGTTCCCCGCATCAACCCCTCCCCGCTTCGGCACCCGTTCC
AAGACGGGTCTGGTGCAGCCGGCAACGTTCCAATTTCGAGAGAAAAAATG
CTGCAGCCGCCCGACTACAAACCAGGATGGTTACAGCCCCTCACAAAAAG
AAGGATAG

**Figure S4.** The ORF of *Zm00001d022394* in WT and *dek504-ref*. The ORF of *Zm00001d022394* produced in WT and *dek504-ref*. The red brackets represent the 6.9-kb deletion in *dek504-ref*. The predicted stop codon of *Zm00001d022394* is indicated in red in WT and *dek504-ref*.

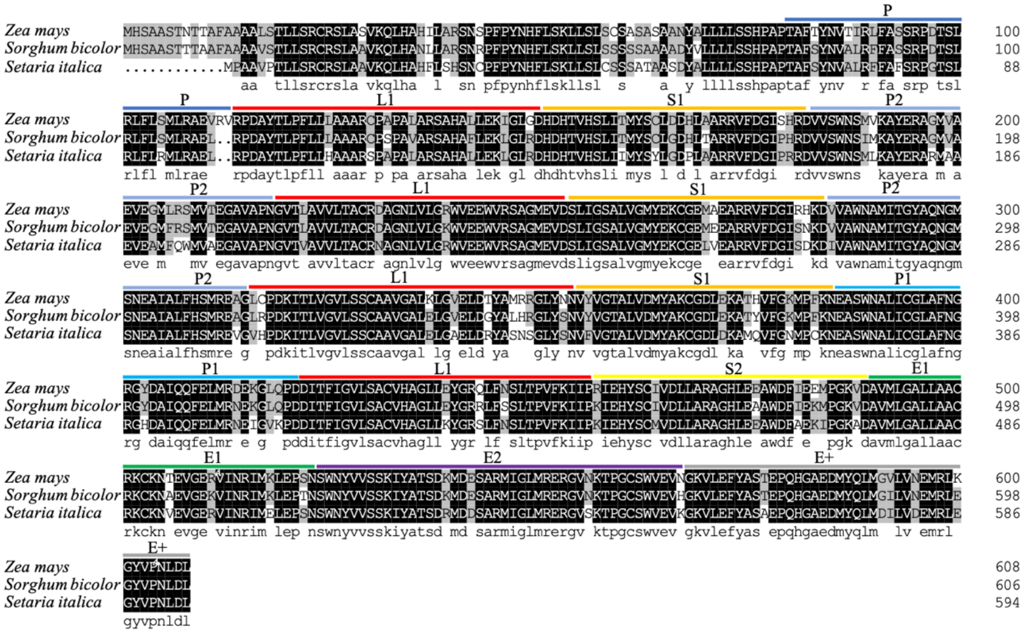

**Figure S5.** Sequence alignment of DEK504 and its homologues from *Sorghum bicolor* and *Setaria italic*. The amino acid sequence of DEK504 homologs, including the *Sorghum bicolor* (XP 021308964.1) and *Setaria italica* (XP 014661047.1), was downloaded from NCBI (https://www.ncbi.nlm.nih.gov/) and was aligned with DNAMAN.

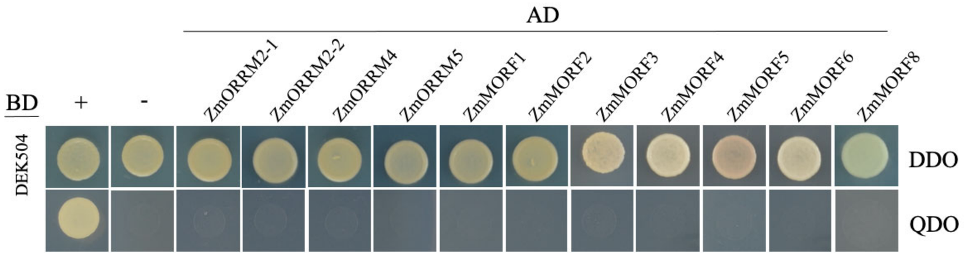


**Figure S6.** A yeast two hybrid (Y2H) assay between DEK504 with organelle RRM proteins (ORRMs) and multiple organelle RNA editing factors/RNA-editing factor interacting proteins (MORFs/RIPs). The colony pictures were taken after three days incubation at 30℃ in SD/- Trp-Leu dropout (DDO) plates, as well as three days incubation at 30℃ in SD/-Trp-Leu-His-Ade dropout (QDO) plates.

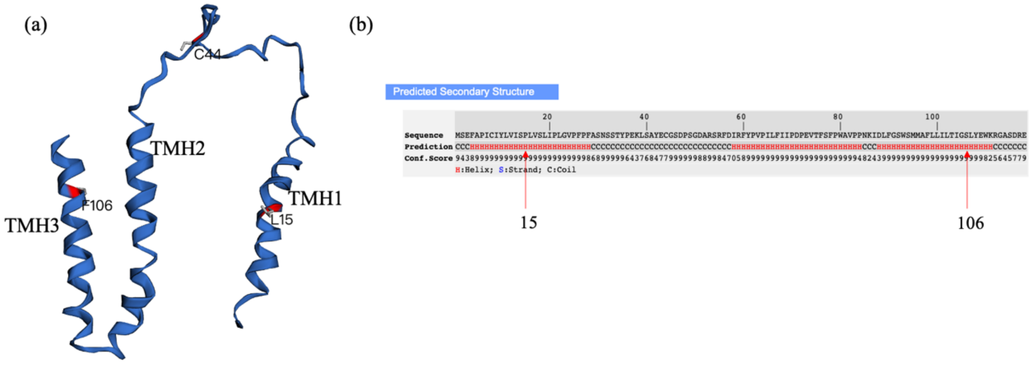


**Figure S7.** Predicted protein structure of NAD3 in the WT. (**a**) The 3D structure was analyzed according to its homology with c6humC_. The L15 and F106 has already been edited, C44 is the important amino acid to access for chemical modification in only the D-form. (**b**) the predicted secondary structure was analyzed by I-TASSER. The amino acids need to be edited is numbered.
